# Psychosocial and Functional Determinants of Central Sensitisation in Patients with Behçet’s Disease: A Cross-Sectional Study

**DOI:** 10.31138/mjr.311025.pfd

**Published:** 2026-06-09

**Authors:** Ayşegül Özdoğan Bircan, Şerife Şeyda Zengin Acemoğlu, Ipek Turk, Didem Arslan

**Affiliations:** Çukurova University, Faculty of Medicine, Department of Internal Medicine, Division of Rheumatology, Adana, Türkiye

**Keywords:** central sensitisation, anxiety, depression, pain, functionality, quality of life

## Abstract

**Objective:**

The purpose of this study was to determine the frequency of central sensitisation (CS) in patients with Behçet’s disease (BD) and to examine its association with disease activity, psychological distress, sleep quality, pain characteristics, and quality of life.

**Methods:**

The study included 99 patients with BD and 67 healthy controls. CS was assessed using the Central Sensitisation Inventory (CSI), with a cutoff score of ≥40. Disease activity was evaluated using the Behçet’s Disease Current Activity Form (BDCAF) and the Behçet’s Syndrome Activity Scale (BSAS). Psychological distress, pain quality, sleep quality, health status, and functionality were measured using the Hospital Anxiety and Depression Scale (HADS), Pain Quality Assessment Scale (PQAS), Pittsburgh Sleep Quality Index (PSQI), Short Form 36 (SF-36), The Health Assessment Questionnaire (HAQ), respectively. Multivariate linear and logistic regression analyses were performed, adjusting for age and gender.

**Results:**

CS was present in 39.4% of BD patients and 3.0 % of controls (p < 0.001). BD patients with CS had significantly higher anxiety, depression, pain severity, disease activity, disability, and sleep disturbance, and lower SF-36 scores (p < 0.001). In multivariate analysis, anxiety and depression were independently associated with higher CSI scores, but only depression remained an independent predictor of CS (OR = 1.486, 95% CI: 1.148–1.923, p = 0.003). The final model explained 41% of CSI variance (adjusted R^2^ = 0.41).

**Conclusion:**

CS in BD is closely linked to psychological factors, independent of disease activity, although causality cannot be inferred from this cross-sectional study.

## INTRODUCTION

Behçet’s disease (BD) is a multisystemic and heterogeneous vasculitis characterised by variable manifestations and of unknown aetiology.^1^ The clinical spectrum may include recurrent mucocutaneous lesions along with neurological, gastrointestinal, and ocular complications.^2^ BD, defined as a relapsing-remitting disease, typically manifests between the ages of 20–40, exhibiting parity in its prevalence among both genders. However, the severity of the disease is found to be more pronounced in male patients.^3^

Central sensitisation (CS) results from high neuronal excitability and increased synaptic transmission triggered by nervous system hyperactivation.^4^ Because of decreased inhibition and aberrant plasticity, increased excitability causes heightened pain sensitivity. ^5^ The three most salient findings of CS are as follows: The association of a painful stimulus with heightened pain (hyperalgesia); the induction of a previously painless stimulus as a source of discomfort (allodynia); and the exacerbated responsiveness to both internal and external stimulants (global sensory hypersensitivity).^5,6^

Neurological involvement occurs in 3% to 30% of BD cases and is a rare and fatal complication.^7^ Clinical manifestations of BD neurological manifestations include headache, dysarthria, fever, paraesthesia, hypoaesthesia, ataxia, urinary incontinence, amnesia, diplopia, nausea, personality changes, psychosis, depression, confusion, seizures, ptosis, erectile dysfunction, cranial nerve palsies, optic atrophy, neuritis, and encephalitis.^8^ Although anxiety and mood disorders, depression, fatigue, sleep disturbance, and sexual dysfunction are associated with neurological involvement, they are frequently observed in patients with BD independently of neurological involvement. These symptoms indicate the presence of a psychiatric burden associated with the disease.^9,10^

CS is a neurophysiological mechanism associated with abnormal pain processing. This mechanism has recently attracted more attention due to its importance in various chronic inflammatory diseases.^11–13^ The presence of CS has been linked to heightened pain intensity, impaired quality of life, sleep disturbances, negative health perception, and increased levels of anxiety and depression. The present study was guided by two over-arching objectives. Primarily, it sought to ascertain the rate of CS in patients with BD. Secondly, it examined the relationship between disease activity, mood disorders, and quality of life. Furthermore, we contend that central pain mechanisms should be incorporated into disease management to facilitate the development of personalised treatment approaches.

## MATERIALS AND METHODS

### Participants and Design

Among 113 patients diagnosed with BD according to the International Classification Criteria,^14^ ninety-nine and 67 age- and sex-matched healthy controls who visited the Çukurova University Rheumatology Clinic between August 2024 and April 2025 were included in the study.

Inclusion Criteria:

- Adults aged between 18 and 75 years at the time of study enrolment.
- Adequate cognitive and physical capacity to complete questionnaire-based assessments.

Exclusion Criteria:

- Below the age of 18 years
- Pregnancy
- Additional rheumatic disease
- Major organ failure
- Presence of concomitant infection
- History of neoplastic disease
- Dementia
- Fibromyalgia
- Psychotropic medication use

This study was approved by the Çukurova University Human Research Ethics Committee (date: July 2024, no: 146/39). All participants were fully informed about the study objectives and procedures. Written informed consent was obtained from all participants prior to enrolment. The study was conducted in accordance with the principles of the Declaration of Helsinki.

### Clinical and Methodological Evaluation Criteria

Information on participants’ demographic characteristics (including age, sex, smoking status, and body mass index), disease duration, and treatment history was systematically recorded.

Two validated instruments, the Behçet’s Disease Current Activity Form (BDCAF) and the Behçet’s Syndrome Activity Scale (BSAS), were used to assess the disease activity of patients with BD. The BDCAF evaluates the presence of new headaches, oral ulcers, genital ulcers, skin lesions, joint involvement, eye involvement, central nervous system involvement, and large vessel involvement within the last four weeks. A score of 0 or 1 is typically assigned to each finding based on its yes/no. The total score ranges from 0 to 12.^15,16^ Unlike the BDCAF, the BSAS is completed by the patient. Consisting of 10 questions, the first six are about mucocutaneous involvement, while the remaining questions address ocular, gastrointestinal, and vascular involvement. Patients are also asked how much each lesion bothers them. The total score ranges from 0 to 100.^17,18^ For this study, active disease was defined as a BDCAF score of at least 4 and a BSAS score of at least 20. This definition was based on clinical relevance and on cut-off values previously used in similar studies.

The Health Assessment Questionnaire (HAQ) was used to evaluate functional ability. The HAQ consists of eight sections and twenty questions. Each question is scored from 0 (I can do it easily) to 3 (I cannot do it at all). The total score is divided by the number of questions answered to determine the final score. A high score indicates low health status.^19,20^

The SF-36 (Short Form 36) Health Survey was used to measure the patients’ health-related quality of life. The questionnaire consists of eight subscales: physical functioning, social functioning, physical and emotional role limitations, general health perception, vitality, mental health, and pain. Each subscale is scored between 0 and 100; higher scores indicate a better health status.^21,22^

The Pittsburgh Sleep Quality Index (PSQI) was used to determine the sleep quality of the patients over the past month. This self-assessment questionnaire consists of 19 items and calculates a total score based on seven components: subjective sleep quality, time to fall asleep, sleep duration, habitual sleep efficiency, sleep disturbances, sleep medication use, and daytime dysfunction. Each component is scored from 0 to 3, resulting in a total score ranging from 0 to 21.^23,24^

The participants’ pain quality was evaluated using the Pain Quality Assessment Scale (PQAS). Twenty items make up this self-assessment scale, which rates different pain feelings from 0 (no pain at all) to 10 (the most excruciating agony imaginable). The 18 questions were divided into three clinically significant subscales based on earlier factor analyses: Paroxysmal pain (shooting, sharp, electric, hot, and radiating), Superficial pain (itchy, cold, numb, sensitive, and tingling), Deep pain (aching, heavy, dull, cramping, and throbbing). For each subscale, subscale scores were calculated by averaging the scores of the relevant items. These subscale scores were used in descriptive and comparative statistical analyses.^25–27^

To assess CS symptoms, the Central Sensitisation Inventory (CSI) was used. It consists of 25 questions, each scored from 0 (never) to 4 (always), resulting in a total score between 0 and 100. A total score of 40 or above is considered indicative of central sensitisation.^28,29^

The Hospital Anxiety and Depression Scale (HADS) was used to assess anxiety and depressive symptoms in the participants. The scale has 14 items altogether, with 7-item subscales for depression (HAD-D) and anxiety (HAD-A). Each subscale has a maximum score of 21, with higher scores denoting more noticeable symptoms. Normal (0–7), borderline (8–10), and abnormal (11 and beyond) were the classifications given to the anxiety and depression subscale scores.^30,31^

### Statistical analysis

**Figure 1.**
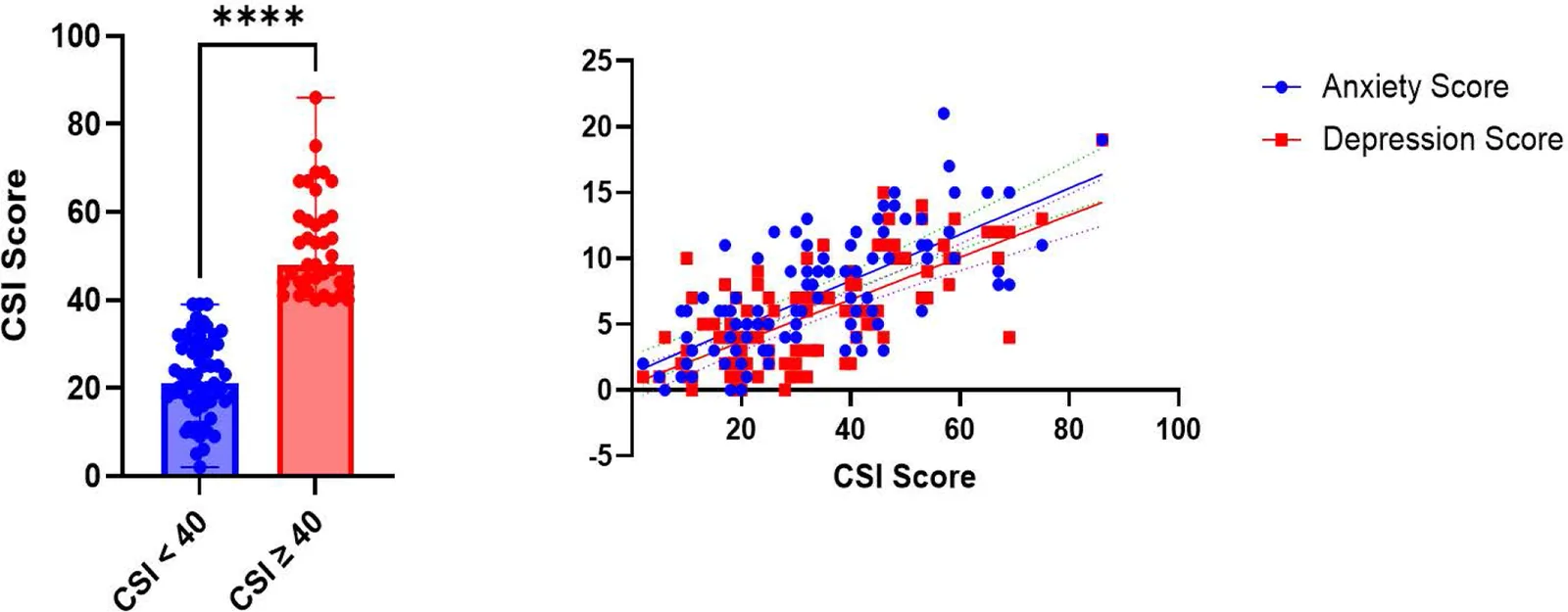
The distribution of CSI scores among patients with and without central sensitisation and the relationships of significant predictors of CSI score.

Data analyses were performed using IBM SPSS Statistics version 27.0 (IBM Corp., Armonk, NY, USA). **Figure 1** was created using GraphPad Prism version 10.4.1 (GraphPad Software, LLC, San Diego, CA, USA). Categorical variables were presented as frequencies (n) and percentages (%), while continuous variables were expressed as median (25th–75th percentile) or mean ± standard deviation, as appropriate. The distribution of data was assessed using both visual methods (histograms and probability plots) and analytical methods (Kolmogorov–Smirnov test). In pairwise comparisons, the Mann–Whitney U test and the unpaired t-test were used for numerical variables, while Pearson’s chi-square test was applied for categorical variables. Post hoc comparisons for multiple categorical variables were conducted using the Z-test for column proportions with Bonferroni correction. Effect sizes for the pairwise comparisons were reported using Cramer’s V and Cohen’s d. Independent variables that showed significant differences between patients with and without CS, and were considered clinically relevant to CSI scores, were included in a multiple linear regression analysis using two models. Multiple linear regression analysis was also performed, adjusting for age and gender. The purpose of employing two models was to identify independent predictors of central sensitisation while controlling for age and gender as potential confounding factors. Assumptions of regression—including linearity, normality of residuals, homoscedasticity, and absence of multicollinearity (defined as VIF < 5 and tolerance > 0.1)—were assessed to ensure the validity of the model. The independent variables included in the multiple linear regression analysis were also entered into a multiple logistic regression model, adjusted for age and gender, to identify risk factors associated with CS. The accuracy and goodness-of-fit of the multiple logistic regression model were assessed using the Hosmer–Lemeshow test, classification tables, and Nagelkerke’s R^2^ statistic. A p-value < 0.05 was considered statistically significant.

### Sample Size Considerations

An a priori power analysis was not performed for sample size calculation, as all eligible patients under clinical follow-up were invited to participate. Post hoc power analyses for the primary outcomes (linear and logistic regression models) were conducted using G*Power software (version 3.1.9.2; Heinrich-Heine-Universität Düsseldorf, Germany), and the achieved statistical power values were calculated and interpreted accordingly.

## RESULTS

Missing data were not present in this study; complete data were obtained for all participants. **Table 1** shows the comparison of demographic and clinical variables between patients with BD and healthy controls. There were no significant differences between the groups in terms of age, gender, smoking status, or comorbidity frequency (p > 0.05). However, patients with BD had significantly lower BMI values compared to controls (p < 0.001). The mean CSI score was significantly higher in BD patients (34.1 ± 17.9) than in controls (28.9 ± 8.7; p = 0.013) (**Figure 1**). Furthermore, the proportion of participants exhibiting CS (CSI ≥ 40) was markedly greater in the BD group (39.4%) compared with controls (3.0%; p < 0.001), indicating a substantially higher prevalence of CS among patients with BD.

**Table 1.** Comparison of demographic and clinical characteristics between patients with Behçet’s disease and healthy controls.

	All Participants n=166	Patients with BD n=99	Controls n=67	p-value

Age, (years)^a^	39.0 (31.0 – 49.0)	39.0 (31.0 – 49.0)	39.0 (33.0 – 49.0)	0.701

Gender, (F)^c^	72 (43.4)	40 (40.4)	30 (44.8)	0.690

BMI, (kg/m^2^)^a^	26.3 (24.1 – 28.7)	25.3 (23.4 – 27.8)	27.0 (24.6 – 28.8)	**0.006**

Current smoking^c^	77 (46.4)	50 (50.5)	28 (41.8)	0.345

Comorbidity^c^	33 (19.9)	17 (17.2)	16 (23.9)	0.288

Type of comorbidity^c^				
HT	21 (12.7)	9 (9.1)	11 (16.4)	0.238
DM	1 (0.6)	0 (0.0)	1 (1.5)	0.223
CAD	1 (0.6)	1 (1.0)	0 (0.0)	0.409
Other	16 (9.6)	11 (11.1)	5 (7.5)	0.435

CSI^b^	35.3±16.9	34.1±17.9	28.9±8.7	**0.013**

CS≥40^c^				
Yes	65 (39.2)	39 (39.4)	2 (3.0)	
				**<0.001**
No	101 (60.8)	60 (60.6)	65 (97.0)	

(a): Median (25% - 75%), b: mean±standard deviation, c: n (%).

BD: Behçet’s Disease; F: Female; BMI: Body mass index: HT: Hypertension; DM: Diabetes mellitus; CAD: Coronary artery disease; CSI: Central Sensitisation Inventory; CS: Central Sensitisation.

The clinical outcomes for patients with and without CS are given in **Table 2**. Patients with CSI scores of 40 or higher exhibited significantly higher levels of anxiety and depression, as well as worse disease activity (BDCAF and BSAS) and higher pain severity across multiple PQAS subdomains, including paroxysmal, deep, and superficial pain (all p < 0.001). Furthermore, these patients reported lower SF-36 scores and higher HAQ scores, as well as poorer sleep quality (PSQI total and subcomponents), compared to patients without CS (all p < 0.01).

**Table 2.** The clinical features, pain characteristics, quality of life, and sleep parameters of Behçet’s disease patients with and without central sensitisation.

	All patients n=99	CSI≥40 n=39	CSI <40 n=60	Effect size (Cramer’s V or Cohen’s d)	p-value

CSI score ^a^	34.5 (21.0 – 45.3)	48.0 (43.5 – 58.0)	25.0 (18.0 – 32.5)	3.12	**<0.001**

Treatment, (Yes) ^c^	91	36	55	0.11	0.909

Type of treatment^c^					
Colchicine	39	31	44	0.07	0.485
MMF	97	37	60	0.18	0.076
Azathioprine	67	24	43	0.11	0.292
Corticosteroids	17	7	10	0.02	0.869

Biological agent^c^					
None	74	31	43		
Infliximab	10	4	6		
Adalimumab	14	3	11	0.19	0.305
Others	1	0	1		

Anxiety score ^a^	6.0 (4.0 – 10.0)	10.0 (7.0 – 13.0)	5.0 (2.3 – 8.0)	1.28	**<0.001**

Anxiety category^c^					
Normal	55	11	44		
Borderline	20	9	11	0.50	<0.001^*^
Abnormal	24	19	5		

Depression score ^a^	6.0 (2.0 – 9.0)	10.0 (6.0 – 12.0)	3.0 (2.0 – 6.0)	1.60	**<0.001**

Depression category^c^					
Normal	68	15	53		
Borderline	16	10	6		
Abnormal	15	14	1	0.55	**<0.001^Ψ^**

SF-36 ^a^					
Physical Function	75.0 (55.0 – 95.0)	60.0 (35.0 – 80.0)	87.5 (70.0 –100.0)	1.17	**<0.001**
Physical Role Difficulty	75.0 (0.0 – 100.0)	0.0 (0.0 – 50.0)	100.0 (0.0 –100.0)	0.95	**<0.001**
Emotional Role Difficulty	100.0 (0.0 – 100.0)	0.0 (0.0 – 100.0)	100.0 (100.0-100.0)	0.89	**<0.001**
Energy/Dynamics/Vitality	45.0 (30.0 – 65.0)	25.0 (15.0 – 40.0)	57.5 (40.0 – 70.0)	1.60	**<0.001**
Mental Health	60.0 (44.0 – 76.0)	44.0 (36.0 – 56.0)	70.0 (57.0 – 80.0)	1.26	**<0.001**
Social Functionality	75.0 (50.0 – 100.0)	50.0 (25.0 – 75.0)	100.0 (65.6 – 100.0)	1.03	**<0.001**
Pain	75.5 (45.0 – 90.0)	45.0 (22.5 – 57.5)	78.8 (55.6 – 90.0)	1.38	**<0.001**
GHP	40.0 (20.0 – 60.0)	25.0 (15.0 – 35.0)	50.0 (35.0 – 63.8)	1.03	**<0.001**

HAQ score ^a^	0.00 (0.00 – 0.47)	0.32 (0.0 – 0.79)	0.00 (0.00 – 0.18)	0.74	**<0.001**

BDCAF score	2.0 (0.0 – 3.0)	2.0 (1.0 – 4.0)	1.0 (0.0 – 2.0)	0.82	**<0.001**

BDCAF category^c^					
Active	17	12	27		
Non-active	82	27	55	0.29	**0.004**

BSAS score ^a^	23.0 (15.0 – 37.0)	31.0 (20.0 – 44.5)	20.0 (11.3 – 32.1)	0.60	**0.004**

BSAS category^c^					
Active	60	30	30		
Non-active	39	9	30	0.27	**0.007**

PQAS score ^a^					
Pain	4.0 (2.0 – 7.0)	6.0 (4.0 – 7.0)	2.5 (0.0 – 5.0)	0.97	**<0.001**
Stabbing	0.0 (0.0 – 3.0)	1.0 (0.0 – 5.0)	0.0 (0.0 – 1.0)	0.64	**<0.001**
Burning	0.0 (0.0 – 2.0)	1.0 (0.0 – 3.0)	0.0 (0.0 – 1.0)	0.50	**0.005**
Distressing	2.0 (0.0 – 5.0)	5.0 (2.0 – 7.0)	0.0 (0.0 – 4.0)	0.84	**<0.001**
Coldness	0.0 (0.0 – 0.0)	0.0 (0.0 – 1.0)	0.0 (0.0 – 0.0)	0.29	**0.016**
Tender	0.0 (0.0 – 1.0)	0.0 (0.0 – 2.0)	0.0 (0.0 – 0.0)	0.28	**0.072**
Crushing	0.0 (0.0 – 3.0)	1.0 (0.0 – 4.0)	0.0 (0.0 – 1.0)	0.43	**0.015**
Itching	0.0 (0.0 – 0.0)	0.0 (0.0 – 1.0)	0.0 (0.0 – 0.0)	0.32	**0.006**
Throbbing	0.0 (0.0 – 4.0)	3.0 (0.0 – 4.0)	0.0 (0.0 – 1.8)	0.78	**<0.001**
Numbness	0.0 (0.0 – 0.0)	0.0 (0.0 – 2.0)	0.0 (0.0 – 0.0)	0.36	**0.016**
Electric-shock-like	0.0 (0.0 – 0.0)	0.0 (0.0 – 2.0)	0.0 (0.0 – 0.0)	0.44	**0.004**
Tingling	0.0 (0.0 – 0.0)	0.0 (0.0 – 2.0)	0.0 (0.0 – 0.0)	0.52	**<0.001**
Cramping	2.0 (0.0 – 3.0)	3.0 (1.0 – 4.0)	0.0 (0.0 – 2.0)	0.72	**<0.001**
Radiating	0.0 (0.0 – 2.0)	1.0 (0.0 – 4.0)	0.0 (0.0 – 1.0)	0.48	**0.007**
Pulsating	0.0 (0.0 – 4.0)	3.0 (0.0 – 5.0)	0.0 (0.0 – 3.0)	0.74	**<0.001**
Aching	0.0 (0.0 – 4.0)	2.0 (0.0 – 4.0)	0.0 (0.0 – 2.8)	0.46	**0.016**
Heaviness	0.0 (0.0 – 4.0)	3.0 (0.0 – 5.0)	0.0 (0.0 – 2.0)	0.81	**<0.001**
Unpleasantness	3.0 (0.0 – 7.0)	6.0 (4.0 – 7.0)	1.0 (0.0 – 5.0)	0.94	**<0.001**
Deep pain	2.0 (0.0 – 5.0)	4.0 (1.0 – 5.0)	0.0 (0.0 – 3.8)	0.67	**<0.001**
Superficial pain	0.0 (0.0 – 2.0)	0.0 (0.0 – 2.0)	0.0 (0.0 – 0.0)	0.34	**0.043**

Paroxysmal Pain ^a^	0.4 (0.0 – 2.2)	1.8 (0.4 – 3.2)	0.0 (0.0 – 1.2)	0.87	**<0.001**
Superficial Pain ^a^	0.0 (0.0 – 1.0)	0.6 (0.0 – 1.3)	0.0 (0.0 – 0.8)	0.61	**0.002**

Deep Pain ^a^	1.7 (0.0 – 4.0)	3.2 (1.6 – 4.7)	0.4 (0.0 – 2.7)	0.89	**<0.001**

PSQI score ^a^					
SSQ	1.0 (1.0 – 2.0)	1.0 (1.0 – 2.0)	1.0 (1.0 – 1.0)	0.58	**0.001**
Sleep Latency	1.0 (1.0 – 2.0)	2.0 (1.0 – 3.0)	1.0 (0.0 – 2.0)	0.74	**<0.001**
Sleep Duration	0.0 (0.0 – 2.0)	1.0 (0.0 – 2.0)	0.0 (0.0 – 1.0)	0.43	**0.020**
HES	0.0 (0.0 – 1.0)	1.0 (0.0 – 1.0)	0.0 (0.0 – 1.0)	0.64	**<0.001**
Sleep Disturbances	1.0 (1.0 – 2.0)	2.0 (1.0 – 2.0)	1.0 (1.0 – 1.0)	0.82	**<0.001**
USM	0.0 (0.0 – 0.0)	0.0 (0.0 – 1.0)	0.0 (0.0 – 0.0)	0.24	**0.026**
Daytime Dysfunction	0.0 (0.0 – 1.0)	1.0 (0.0 – 2.0)	0.0 (0.0 – 1.0)	0.79	**<0.001**
Total PSQI Score	5.0 (3.0 – 9.0)	9.0 (5.0 – 12.0)	4.0 (2.0 – 7.0)	1.10	**<0.001**

(a): Median (25% - 75%), c: n (%),

(*): p<0.05 for normal and abnormal category,

(Ψ): p<0.05 for normal, borderline, and abnormal category.

CSI: Central Sensitisation Inventory; MMF: Mycophenolate mofetil; SF-36: Short Form 36; GHP: General Health Perception;HAQ: Health Assessment Questionnaire; BDCAF: Behçet's Disease Current Activity Form; BSAS: Behçet's Syndrome Activity Scale; PQAS: Pain Quality Assessment Scale; PSQI: Pittsburgh Sleep Quality Index;SSQ: Subjective Sleep Quality;HES: Habitual Sleep Efficiency; USM: Use of Sleep Medication.

After adjusting for age and gender, a multiple linear regression analysis revealed that anxiety and depression scores were significant predictors of CSI scores. Anxiety and depression were positively associated with central sensitisation, increasing CSI scores by 0.99 and 1.61 points per unit increase in anxiety and depression scores, respectively. However, disease activity (BDCAF and BSAS) and pain subtype scores were not significant predictors. The final model explained a substantial proportion of the variance in CSI scores, with an adjusted R^2^ of 0.41, indicating that approximately 41.0% of the variance with 100% power (f^2^ = 1.84, corresponding to R^2^ = 0.648) in CS levels could be accounted for by the included predictors beyond the effects of age and gender (**Table 3**, **Figure 1**).

**Table 3.** Multiple linear regression analysis of clinical and psychosocial predictors of central sensitisation inventory score adjusted for age and gender.

Model 1	B (SE)	95%CI for B	Beta	VIF/Tolerance
Age	0.06 (0.15)	−0.23 to 0.35	0.036	1.01 / 0.99
Gender (F)	17.11 (3.27)^*^	10.61 to 23.61	0.471	1.01/0.99
Model 2				
Age	0.02 (0.11)	−0.19 to 0.23	0.013	0.78/1.29
Gender (F)	10.14 (2.58)^*^	5.03 to 15.27	0.279	0.92/1.08
Anxiety score	0.99 (0.41)^*^	0.18 to 1.80	0.249	0.37/2.72
Depression score	1.61 (0.45)^*^	0.72 to 2.50	0.365	0.38/2.62
BDCAF score	0.80 (1.09)	−1.37 to 2.97	0.072	0.40/2.51
BSAS score	0.10 (0.11)	−0.13 to 0.32	0.085	0.40/2.53
Paroxysmal Pain	1.04 (1.14)	−1.24 to 3.32	0.099	0.33/3.03
Superficial Pain	−1.30 (1.76)	−4.79 to 2.19	−0.740	0.39/2.56
	Model 1		Model 2	
Note	Constant (SE)=24.87 (6.13)		Constant (SE)=7.80 (5.15)	
	Adjusted R^2^= 0.23		R2 change=0.41	

B: unstandardised coefficients; SE: standard error; β: standardised coefficients; F: Female; BDCAF: Behçet's Disease Current Activity Form; BSAS: Behçet’s Syndrome Activity Scale

(*) p < 0.05.

**Figure 2.**
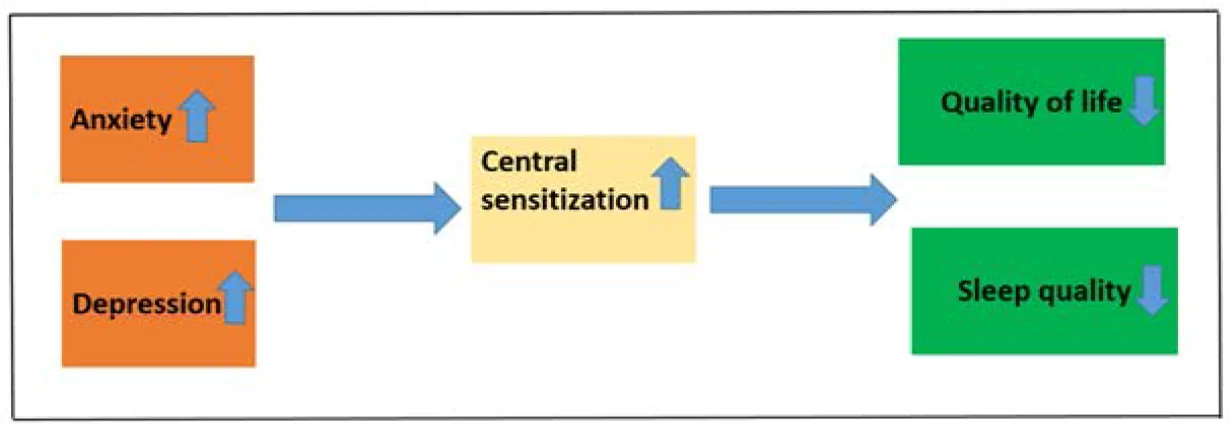
Conceptual path diagram illustrating relationships among anxiety, depression, central sensitisation, sleep quality, and quality of life.

The conceptual path diagram illustrating the effects of anxiety and depression on CSI scores, as well as the reduced quality of life and sleep quality observed in patients with CS, is presented in **Figure 2**.

**Table 4** presents the results of a multivariable logistic regression analysis that assessed the association between clinical and psychosocial variables and the presence of central sensitisation after adjusting for age and gender. Of the included predictors, only the depression score was significantly associated with an increased likelihood of CS (odds ratio [OR] = 1.486, 95% confidence interval [CI]: 1.148–1.923, p = 0.003) with low (1-B = 0.46) power. This suggests that for each one-point increase in the depression score, the odds of exhibiting central sensitisation increase by approximately 49%. However, other variables, including anxiety, disease activity scores (BDCAF and BSAS), and specific pain characteristics (paroxysmal and superficial pain), were not significantly associated with central sensitisation (p > 0.05 for all).

**Table 4.** Multivariable logistic regression analysis of factors associated with central sensitisation, adjusted for age and gender.

Variable	B (SE)	OR (95% CI)	Wald
Anxiety score	0.55 (0.11)	1.056 (0.857 to 1.303)	0.263
Depression score	0.40 (0.13)^*^	1.486 (1.148 to 1.923)	9.062
BDCAF score	0.37 (0.28)	1.449 (0.842 to 2.497)	1.791
BSAS score	−0.02 (0.03)	0.982 (0.925 to 1.042)	0.368
Paroxysmal Pain	0.00 (0.26)	1.002 (0.598 to 1.680)	0.000
Superficial Pain	0.10 (0.42)	1.109 (0.489 to 2.513)	0.061

Model statistics: Nagelkerke R^2^ = 0.56; Hosmer–Lemeshow χ^2^(8) = 10.15, p = 0.26.

B: Unstandardised coefficient; SE: Standard error; OR: Odds ratio; BDCAF: Behçet's Disease Current Activity Form; BSAS: Behçet’s Syndrome Activity Scale.

(*) p < 0.05.

## DISCUSSION

This study investigated the rate of CS in patients with BD and its association with clinical and psychosocial factors. In our study, 39.4% of BD patients and 3.0% of healthy controls exhibited CS. An analysis involving 88 patients with BD and 60 healthy controls found that CS was more frequent and occurred at a higher rate in the BD group than in healthy controls (69.3% vs. 28.3%, respectively, p<0.001). ^32^ In line with our findings, the CS rate was higher in the patient group. However, a more prevalent CS was observed in both groups compared to our study.

A major finding of our study was that patients with BD who experienced CS exhibited significantly diminished sleep quality, elevated anxiety and depression levels, heightened pain intensity, exacerbated disease activity, inferior health perception, and lower quality of life in comparison with patients without CS. The current results emphasise the importance of considering central pain mechanisms in the clinical evaluation and management of BD, particularly in patients experiencing abnormal pain or functional impairment. There is a substantial correlation between CS and a number of outcomes, such as depression, anxiety, stress, pain catastrophising, sleep problems, and kinesiophobia, according to a meta-analysis of 66 research with 13,284 participants. The same analysis emphasises that self-report questionnaires, such as the CSI, do not measure the neurophysiological mechanisms of CS. Rather, they measure an individual’s psychological sensitivity and anxiety toward pain or bodily signals. Therefore, it is argued that these questionnaires are limited in their ability to measure biologically-based CS.^33^ Our work emphasises the important role of depressive symptoms on CS in BD, reflecting findings in patients with primary Sjögren’s syndrome, where psychological factors such kinesiophobia and poor sleep quality were connected to higher fear-related outcomes. ^34^

In multivariate analysis, BDCAF and BSAS scores did not have independent predictive significance, despite being higher in the CS-positive group. Our results were in line with the literature on the topic, which found that pain centralisation is a feature that contributes to pain irrespective of the effects of inflammation in a research that involved 263 individuals with rheumatoid arthritis.^35^ The prevalence of CS in patients with primary Sjögren’s syndrome was similarly linked to the subjective disease activity indicator known as the EULAR Sjögren’s Syndrome Patient Reported indicator (ESSPRI), according to another study. The EULAR Sjögren’s Syndrome Disease Activity Index (ESSDAI), an objective clinical scoring system, did not, however, demonstrate a significant correlation with the study. CS may be a patient-reported burden that is unrelated to disease activity, according to these data. ^11^

Numerous studies have demonstrated a significant correlation between CSI scores and various quantitative sensory testing (QST) methods. This supports the idea that the scale can accurately reflect central pain processing.^36^ Pain prevalence mirrored pain intensity but did not clearly indicate the presence of CS, according to a study which investigated the relationship between pain prevalence and CS symptoms in 30 female fibromyalgia patients as determined by QST. ^37^ In order to assess potential sensory traits that might be connected to CS, we examined the paroxysmal, deep, and superficial pain subtypes independently. All of these pain kinds did not, however, significantly correlate with CS in the multivariate analysis. The idea that CS may develop based on a variety of circumstances, not only the type of pain, is supported by this finding.

Pain and sleep are connected in a vicious cycle. Pain can disrupt sleep, and poor sleep quality can lower pain thresholds and amplify painful sensations.^38^ 67–88% of chronic pain problems include insomnia, and at least 50% of people with insomnia the most prevalent sleep disorder also have chronic pain.^39^ Our findings support the literature, showing that sleep disorders were significantly associated with all components of CS in the group.

As our study predicted, the mental and physical components of the SF-36, as well as overall health perception, were significantly affected in patients with CS. CS was found in 47.5% of 80 patients with chronic shoulder pain in a cross-sectional investigation. It was adversely connected with SF-36 components and favourably connected with disability. ^40^

CS is strongly linked to somatic symptoms, anxiety, and depression, according to recent research.^41,42^ According to our findings, patients with CS had greater rates of anxiety and depression, while only depression scores were independently associated with CS in the multivariate analysis. In a recently published study examining the prevalence of CS and neuropathic pain (NP) in 114 patients with psoriatic arthritis and a median disease duration of four years, our findings were supported by the observation that anxiety and fibromyalgia were more prevalent in patients with CS and NP, while depression was only more prevalent in patients with CS. ^43^ We think that the following factors may be responsible for our findings: Depression has a greater impact on pain perception and the emotional burden of pain than other variables; depression is linked to limbic system dysfunction, which directly affects central pain modulation; depressed people have a lower pain threshold and are more sensitive to sensory stimuli. It accounted for almost 41% of the variance in CSI ratings in our age- and gender-adjusted multiple linear regression analysis (adjusted R2 = 0.41). The idea that psychological and clinical factors, especially depression, contribute significantly to CSI variance is supported by this modest explanatory power.

There are a number of limitations to this investigation to take into account. First, the cross-sectional design prevents us from establishing causal relationships between CS and clinical or psychosocial variables. As a result, the study’s findings should be viewed as generating hypotheses rather than as causal. Longitudinal studies are necessary for this determination. Second, the CSI, a widely used screening tool, does not directly measure neurophysiological mechanisms of CS and may be influenced by psychological characteristics, such as somatisation and catastrophising. The absence of QST, which is considered the gold standard for assessing CS, and the exclusive reliance on the self-reported CSI questionnaire represent important limitations of the study in terms of validation. Future studies incorporating QST or other objective assessment methods are warranted to provide objective confirmation and strengthen the validity of the current findings. Finally, our study did not assess some potential confounders, such as neuroimaging, cytokine levels, and inflammation markers. Additionally, the low power (1-β = 0.46) obtained in the logistic regression analysis indicates that further studies with larger sample sizes are required to confirm this finding.

In conclusion, this study emphasises the significance of CS in patients with BD and reveals depression’s predominant role. Although disease activity measures were not found to be independently associated with CS, subjective symptoms such as mood disorders, sleep disorders, increased pain intensity, and decreased quality of life showed strong associations. These results imply that central mechanisms, particularly psychological factors, may contribute to persistent pain and symptom burden in BD beyond inflammatory activity. These findings emphasise the importance of psychosocial assessments in the clinical management of CS in Behçet’s disease patients. Routine screening for depression, anxiety, and sleep disorders, in particular, may contribute to the early identification of factors that perpetuate central pain mechanisms. Non-pharmacological interventions targeting CS—cognitive behavioural therapy, education about sleep hygiene, exercise-based approaches, and pain education—may be useful in reducing symptom burden. Furthermore, a multidisciplinary approach involving rheumatology, psychiatry, physical therapy, and pain management units may offer a more effective option for improving both pain control and patient quality of life.

## Data Availability

The datasets gathered during the preparation of this manuscript are available from the corresponding author upon reasonable request.
